# Development and evaluation of a novel prehospital antidote service providing methylthioninium chloride (methylene blue) for sodium nitrite poisoning

**DOI:** 10.1136/emermed-2024-214777

**Published:** 2025-10-08

**Authors:** Gregory Davies, Jason Wiles, Alison Walker, Christopher Humphries

**Affiliations:** 1 West Midlands Ambulance Service NHS Foundation Trust, Brierley Hill, England, UK; 2 Emergency Department, Harrogate and District NHS Foundation Trust, Harrogate, UK; 3 Centre for Cardiovascular Science, The University of Edinburgh Queen’s Medical Research Institute, Edinburgh, Scotland, UK; 4 Toxicology, Addictions and Mental Health Emergencies (TAME) Research Group, The University of Edinburgh Queen’s Medical Research Institute, Edinburgh, Scotland, UK

**Keywords:** pre-hospital care, toxicology, extended roles, effectiveness, deliberate self

## Abstract

**Background:**

Sodium nitrite has become established as a method of self-harm and suicide. Toxicity occurs primarily through the formation of methaemoglobin (MetHb). In response to a coroner request, West Midlands Ambulance Service developed a prehospital methylthioninium chloride (methylene blue) capability within the specialist Hazardous Area Response Team (HART) to treat methaemoglobinaemia. A service evaluation was planned to understand the impact.

**Methods:**

A retrospective observational series of patients, during a planned evaluation period from 1 July 2020 to 1 July 2024. All patients receiving a HART response for suspected sodium nitrite poisoning were included. A Patient Group Direction (PGD) for the treatment of methaemoglobinaemia by paramedics was produced, allowing treatment with intravenous methylthioninium chloride 1–2 mg/kg in specific circumstances. MetHb levels were assessed using handheld pulse CO-Oximeters.

**Results:**

Nine patients were attended for suspected sodium nitrite toxicity and three were administered prehospital methylthioninium chloride under PGD indications. In addition, one patient had no evidence of toxicity, four had conditions unequivocally associated with death and one was in cardiac arrest. The patient in cardiac arrest received sodium nitrite in hospital from the HART team under verbal direction from medical staff but did not survive. Serial CO-Oximeter readings for two of the three patients who received methylthioninium chloride indicated a decrease in MetHb levels and all three survived. Consumable costs associated with the new capability were minimal. Potential improvements to the service were identified, including changes to PGD indications.

**Conclusion:**

The limited number of cases seen in the evaluation period suggests that restricting the prehospital provision of methylthioninium chloride to specialist teams is proportionate, and it appears feasible for specialist paramedics to deliver prehospital methylthioninium chloride under PGD indications. However, prehospital use of methylthioninium chloride for the treatment of methaemoglobinaemia currently lacks sufficient evidence to support widespread adoption without further evaluation.

**WHAT IS ALREADY KNOWN ON THIS TOPIC**

- Sodium nitrite is a potent toxin which may be used as a method of suicide to induce methaemoglobinaemia. Earlier treatment may improve outcomes following ingestion.
- The antidote in routine use for methaemoglobinaemia is methylthioninium chloride (methylene blue), which is currently only routinely available within hospitals in the UK. The feasibility of a paramedic-delivered methylthioninium chloride service is not known.

**WHAT THIS STUDY ADDS**

- This evaluation demonstrates that it is feasible to introduce a novel prehospital antidote capability for the treatment of methaemoglobinaemia following suspected sodium nitrite ingestion. This capability led to nine patient attendances, three instances of patients receiving treatment in the prehospital environment and one case of in-hospital treatment.
- The data presented demonstrate an improvement in methaemoglobin levels temporally associated with the administration of prehospital methylene blue.

**HOW THIS STUDY MIGHT AFFECT RESEARCH, PRACTICE OR POLICY**

- The feasibility of the service presented here may lead to national adoption of similar models of care. If widespread adoption occurs, further evaluation (such as a stepped wedge cluster randomised trial) would be warranted as a critical part of implementation.

## Introduction

Sodium nitrite is an inorganic compound, historically best known to clinical toxicologists as an antidote with a role in the treatment of cyanide toxicity.^1 2^ While it is still licensed for this purpose, and has legitimate public uses, including as a meat preservative and as a corrosion inhibitor, data have emerged in recent years suggesting that it has become an increasingly well-known method of deliberate self-harm ingestion and suicide.^3^ Discussion of the use of sodium nitrite as a method of suicide on online forums has increased, and though the UK lacks robust data on self-harm poisonings, it appears that use of sodium nitrite as a method of suicide may be growing.^4 5^

The mechanism of sodium nitrite toxicity is through the conversion of haemoglobin to methaemoglobin (MetHb), by indirect oxidation of ferrous iron to ferric iron which cannot bind oxygen, leading to tissue hypoxia and oxidative stress.^6^ Nitrite-induced vasodilation may also occur, contributing to hypotension. The typical antidote provided in hospitals is methylthioninium chloride (methylene blue), which acts as an electron donor to reduce MetHb.^7^ While recommendations regarding antidote stocking for emergency departments exist, the only antidotes routinely available in the UK prehospital setting are activated charcoal, atropine and naloxone.^8 9^ Given the time-critical nature of these presentations, and the potential for the peak effect of methylthioninium chloride to be delayed for up to 30 min after administration, there have been calls to introduce methylthioninium chloride to the prehospital environment.^10^

UK coroners may issue a Regulation 28 Prevention of Future Deaths Report to specific individuals or organisations, when they believe that action should be taken to prevent future deaths. West Midlands Ambulance Service (WMAS) received such a report in January 2020, requesting an examination of ways to improve patient outcomes following sodium nitrite ingestion, and specifically examining the role of methylthioninium chloride.

A pilot programme was developed to allow the WMAS Hazardous Area Response Teams (HART) to provide methylthioninium chloride for the treatment of methaemoglobinaemia under a Patient Group Direction (PGD).^11^ PGDs allow a legal framework for the supply and administration (by healthcare professionals) of specified medicines to predefined patient groups in prespecified circumstances. This allows the provision of treatments without the case-by-case input of a prescriber. A service evaluation was planned to understand the impact of the change on clinical effectiveness and prehospital clinical outcomes (in all patients assessed for the potential administration of methylthioninium chloride) and resource utilisation (at a service level). The results of the evaluation and case series of patients treated are presented here.

## Methods

### Design and participants

The study design was a retrospective observational series of all patients receiving a HART response for suspected sodium nitrite poisoning from 1 July 2020 to 1 July 2024.

### Setting

WMAS is an ambulance service provider (emergency medical service—EMS—provider) to a population of 5.6 million, over an area of 5000 square miles. UK ambulance services all have HART units tasked with providing the response in the presence of potentially hazardous materials or environments, and the provision of specialist skills, equipment and medications not provided by standard emergency ambulances.

Incidents featuring suspicious substances (eg, an unidentified white powder, later found to be sodium nitrite) automatically trigger a WMAS HART response. If the presence of sodium nitrite only became apparent after the initial call (eg, on standard crew attendance), a HART response would then become indicated. Interrogation by the Incident Command Desk was a parallel process to standard EMS dispatch, and HART dispatch therefore did not delay routine clinical care if attendance by a non-specialist crew was appropriate based on initial information.

Of note, if initial call information suggests an unwell patient in the context of a suspicious substance, it is standard care that a routine EMS crew would not commence clinical care due to the potential risk to care providers and would instead await the arrival of HART. However, if clinical care has already commenced, and ingestion of sodium nitrite subsequently becomes apparent, routine EMS crews may transport the patient to hospital without awaiting HART. In these cases, HART would typically attempt to intercept the patient in transit to assist in providing clinical care. In cases of suspected sodium nitrite ingestion where the victim has unequivocal signs of death, HART will be called but will not treat. These calls may have slower response times given the lack of urgency in these situations.

Methylthioninium chloride was stocked as 50 mg/10 mL ampoules for injection. MetHb percentage values were obtained using Masimo Rad-57 Handheld Pulse CO-Oximeters with Masimo Rainbow SET Technology. PGD-specific training to WMAS HART paramedics was provided as part of standard training cycles. All patients in whom there was a clinical suspicion of methaemoglobinaemia had CO-Oximetry performed.

The indications for treatment of methaemoglobinaemia were based on the WMAS interpretation of TOXBASE advice, applied to the prehospital setting at the time when the PGD was authorised. TOXBASE is the clinical toxicology database of the UK National Poisons Information Service (NPIS), accessible by health professionals as an additional support tool for medical decision-making.^6^ Adaptation was primarily aimed at delivering a pragmatic treatment guideline for use in the prehospital setting to reduce risk for patients who were likely to be at the greatest risk of poor outcomes. Indications for treatment of methaemoglobinaemia under the PGD are provided in table 1.

**Table 1:** Indications for using methylthioninium chloride in the treatment of methaemoglobinaemia under the West Midlands Ambulance Service Hazardous Area Response Team (WMAS HART) Patient Group Direction (PGD)

	WMAS HART
**Indications (any of**)	Life-threatening cases even if point-of-care MetHb unavailable. MetHb with evidence of end-organ dysfunction (eg, shortness of breath, chest pain, confusion). Consider if MetHb <30% with pre-existing disease increasing susceptibility to tissue hypoxia.
**Dosage**	1–2 mg/kg
**Maximum dosage**	Repeat dose at 60 min in cases of persistent or recurrent symptoms, or if MetHb levels remain significantly higher than normal. Maximum 4 mg/kg total.
**Method of administration**	5 mg/mL concentration from ampoule by slow intravenous injection (5 min).
**Role in cardiac arrest**	Not indicated under PGD.

Methaemoglobin levels are using portable CO-Oximetry.

MetHb, methaemoglobin.

### Data collection and analysis

Patient data collected were limited to data contained within electronic patient record forms. All domains for which data were collected are reported in the results without omission. Data collection was undertaken retrospectively by all evaluators (GD, JW, AW, CH) and consensus on any areas of data abstraction disagreement was reached through group discussion. In-hospital data (eg, venous blood gas results) were not available.

Costs assessed were those additional costs directly attributable to the acquisition of consumables for the HART capability to provide methylthioninium chloride antidote therapy, rather than the entire cost attributable to responding to the patient. Costings were provided directly by WMAS HART and are provided as British Pound Sterling (GBP), excluding Value Added Tax (VAT), rounded to the nearest whole number.

### Patient and public involvement

Patient and public involvement was not sought for this service evaluation. Introduction of the service was as a result of a coroner’s request requesting that WMAS examine the role of methylthioninium chloride in treating sodium nitrite ingestion.

## Results

**Figure 1:**
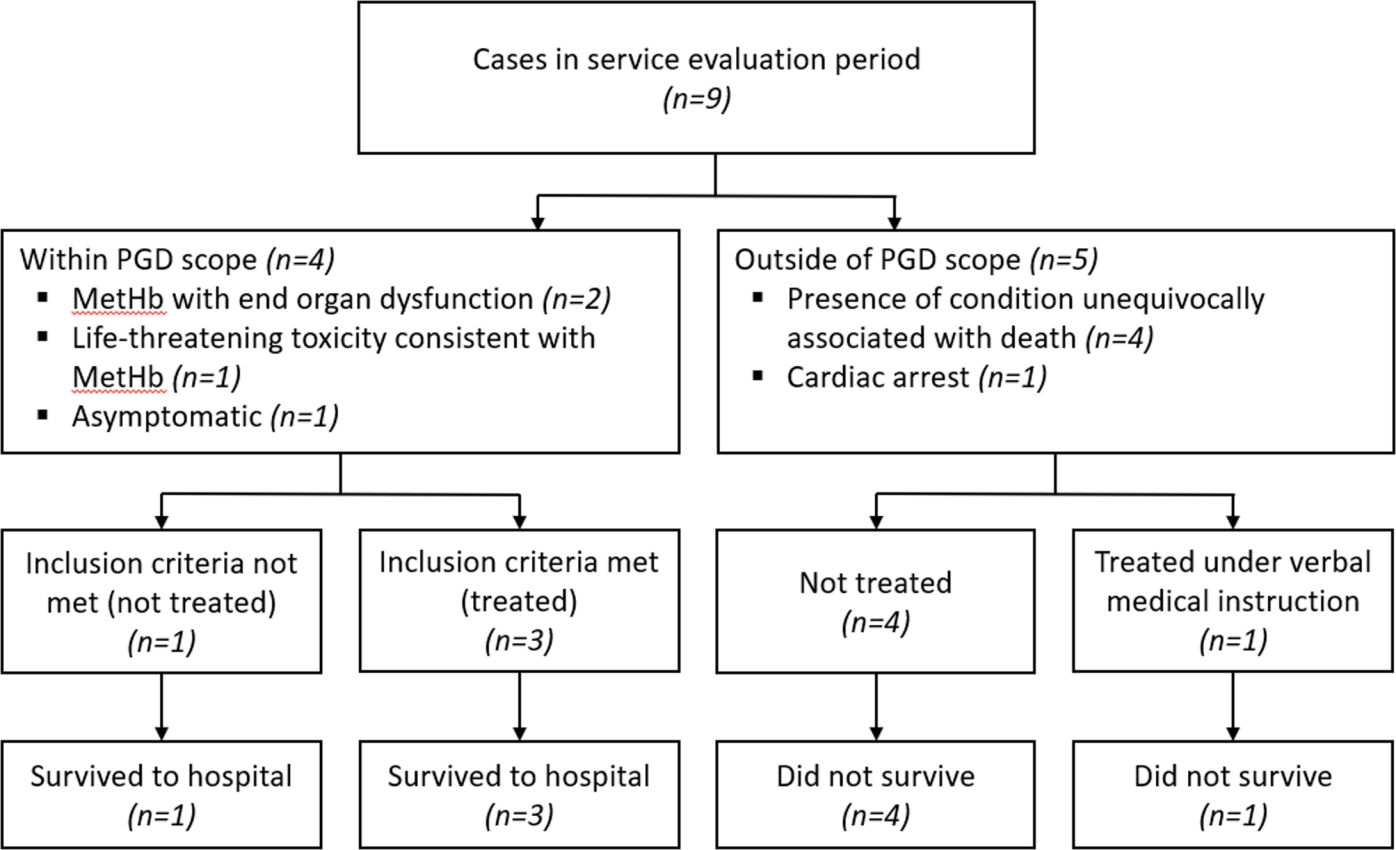
Service evaluation flow chart. Conditions unequivocally associated with death are: massive cranial and cerebral destruction, hemicorporectomy or similar massive injury, decomposition/putrefaction, incineration, hypostasis, rigour mortis. MetHb, methaemoglobin; PGD, Patient Group Direction.

Nine cases received a HART response for sodium nitrite ingestion. Four patients had conditions unequivocally associated with death and were not treated (figure 1). Three patients received methylthioninium chloride under PGD eligibility criteria. One additional patient reported sodium nitrite ingestion but had no clinical findings suggestive of toxicity and a normal MetHb percentage on CO-Oximetry and therefore did not receive methylthioninium chloride. A further patient was in cardiac arrest and thus ineligible under the PGD (identified as the patient in cardiac arrest in figure 1); this patient received treatment with methylthioninium chloride for clinically suspected sodium nitrite ingestion from HART under the explicit direction of medical staff on arrival at hospital as an adjunct to cardiopulmonary resuscitation and did not survive (dose not recorded).

**Figure 2:**
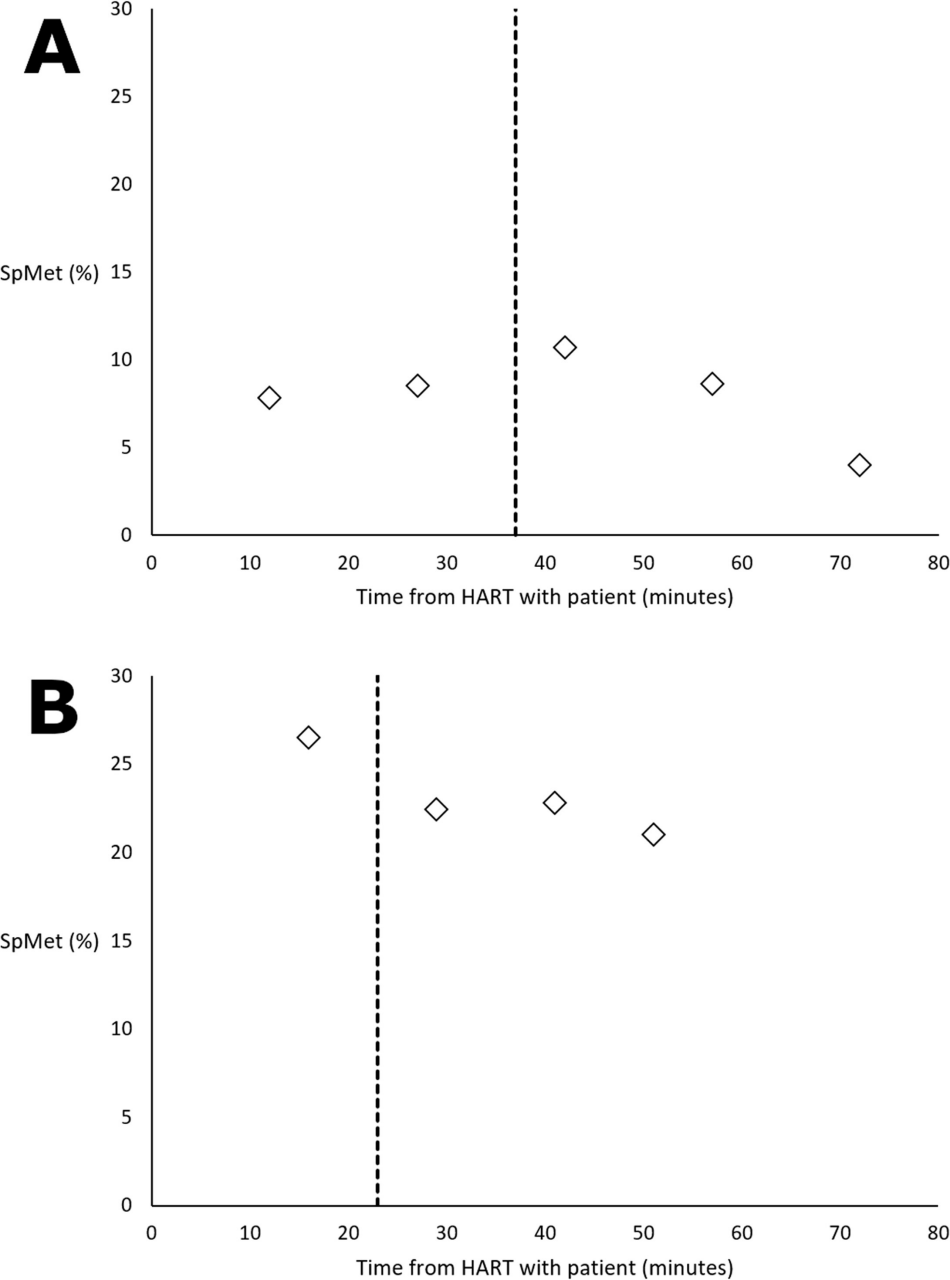
SpMet (%), as measured by a handheld pulse CO-Oximetry, in (**A**) patient 1 and (**B**) patient 2, before and after methylthioninium chloride administration (indicated by the vertical dashed line). HART, Hazardous Area Response Team; SpMet, methaemoglobin saturation.

Patients 1 and 2 had clearly reported times of ingestion and methylthioninium chloride dosing, as well as serial MetHb readings documented during treatment and these are therefore included for interest (figure 2). Patient 3 had clinical findings consistent with sodium nitrite toxicity, but poor peripheral perfusion precluding CO-Oximetry.

Table 2 describes the five patients assessed for prehospital administration of methylthioninium chloride (four under PGD indications, one under verbal instruction). The data demonstrate variation within each time-stamped aspect of the patient journey, though the time from HART arrival to antidote use became shorter with increasing HART experience. Improvements were seen in SpMet readings in patients 1 and 2 following treatment, while readings could not be obtained in the other treated patients (3 and 5).

**Table 2:** Timings, clinical findings and prehospital outcomes of patients with suspected sodium nitrite-induced methaemoglobinaemia assessed for potential prehospital administration of methylthioninium chloride

	Patient 1	Patient 2	Patient 3	Patient 4	Patient 5
**Gender**	Female	Female	Male	Male	Male
Age (years)*	30–39	30–39	30–39	50–59	10–19
**Self-reported ingestion (g**)	25	30	9	Not reported—substance evident on scene	50
**Clinical findings**	Respiratory distress, tachycardia, reduced level of alertness, diaphoresis	Respiratory distress, tachycardia, chest discomfort	Respiratory distress, tachycardia, chest discomfort, diaphoresis, confusion	No evidence of end-organ dysfunction	Cardiac arrest
**SpMet (%**)	10.7	26.5	Unrecordable (poor peripheral perfusion)	0	N/A
**Antidote dose (mg**)	70	70	90	N/A	Not recorded
**Lowest subsequent SpMet (%**)	4.0	21.0	Not available	N/A	N/A
**Initial call to HART activation (minutes**)	15	43	25	68	16
**HART activation to with patient (minutes**)	30	16	7	18	44
**HART with patient to antidote use (minutes**)	37	23	22	N/A	0
**Antidote use to hospital arrival (minutes**)	44	30	22	62	N/A
**Outcome at hospital arrival**	Survived to hospital	Survived to hospital	Survived to hospital	Survived to hospital	Cardiac arrest

HART, West Midlands Ambulance Service Hazardous Area Response Team.

Patient 4 was not treated, and therefore no antidote use timestamp is reported. Patient 5 was in cardiac arrest: HART were unable to intercept the patient in transit to the hospital, and therefore met the crew on arrival at the receiving hospital with immediate treatment. Consequently, no hospital arrival timestamp is reported.

* Ages are reported as ranges to support pseudonymisation—all patients were adults.

N/A, not assessed; SpMet, methaemoglobin saturation.

Consumables costs associated with the introduction of methylthioninium chloride were minimal. Two of the CO-Oximeters used were already stocked on each HART vehicle to meet other service capability requirements. If a novel service required purchasing these devices, provision of a single device would cost approximately £5825. Supplying all three HART vehicles with methylthioninium chloride had an initial cost of £193 per vehicle (three vehicles) and a per-dose consumables cost of approximately £80 for a 1 mg/kg adult dosing.

## Discussion

### Feasibility

This service evaluation demonstrates that the pathway was not only technically deliverable by specialist paramedics, but also workable as a wider service. The findings support the feasibility of the overall service model from clinical, governance and logistical perspectives.

A key finding is the successful implementation and use of the PGD. This demonstrates governance feasibility, providing a robust legal and clinical framework for a complex, high-risk and infrequent intervention in the prehospital environment. Feasibility was also evident in the paramedics’ clinical decision-making; three eligible patients were identified, while treatment was appropriately withheld from a patient with no objective evidence of toxicity, clearly demonstrating professional capacity for nuanced clinical judgement, not just task completion.

Furthermore, the data support the proportionality and sustainability of the model. The low number of cases confirms that restricting this specialist capability to HART is an appropriate and efficient use of resources. This targeted approach is made economically feasible by leveraging existing HART infrastructure, such as the pre-existing stock of CO-oximeters and the integration of new competencies into routine training cycles. In contrast, a service expansion to all routine ambulances would likely be prohibitively expensive and face significant logistical barriers related to medicine storage and governance, reinforcing that this specialist team model is a pragmatic and workable solution.

### Time to HART activation, treatment and transport

The duration between initial call and HART activation can be significant. This suggests that there may be work to be done on appropriate flags to expedite the identification of potential cases to HART dispatch systems, but this would require amendments by National Health Service Pathways and Medical Priority Dispatch call-taking systems. Callers may not use the chemical name for sodium nitrite, but may use colloquialisms describing types of salt, such as ‘curing salt’.^10^ Inevitably, in some cases, evidence of sodium nitrite ingestion will only become apparent after the initial call—either due to evidence on scene or clinical interrogation of the caller.

Time from HART activation to arrival is a feature of geography and may be seen as introducing unnecessary delay. However, the authors would emphasise that in some contexts (eg, when it is known from call information that there is an acutely unwell patient in the context of an unknown substance), HART is considered the only appropriate dispatch response due to the potential risk to care providers and their attendance does not delay a standard ambulance attendance, as this would not be provided. Additionally, in cases where clinical care had already commenced prior to sodium nitrite ingestion being identified, no standard EMS crew were prevented from transporting their patient to hospital if they felt able to do so (as seen with patient 5, where HART attempted to intercept the patient in transit, but were unable to do so, and therefore met the patient on arrival at hospital).

Time from HART arrival to treatment was longest with patient 1. The HART members involved acknowledged a steep learning curve requiring senior clinical advice, and improvements were seen in time to treatment in subsequent cases. While this may appear to be a delay which would not occur in hospital, the most recent available UK audit data suggests that only 84.3% of hospitals have methylthioninium chloride available immediately.^12^ This was borne out in HART’s clinical experience; the patient not treated under the PGD as they were in cardiac arrest in fact had methylthioninium chloride administered by HART within a hospital resuscitation room on a doctor’s verbal instruction, as the receiving hospital was unable to locate their own antidote stock.

Time from treatment to arrival at hospital is not considered to represent an additional delay versus non-HART care.

### Patient outcomes

To our knowledge, all patients who survived to hospital arrival subsequently survived to discharge, as there have been no coroner’s inquests relating to these cases. The authors acknowledge the small patient numbers in this observational series limit claims regarding treatment benefits and that there is the potential for unidentified consequences. Nevertheless, the centralisation of care to a specialised resource does not appear to have negatively impacted any clinical outcomes in the prehospital phase of patient care, though the study lacks a control group.

### Quality of data

Due to the non-standard nature of sodium nitrite cases triggering call transfer to the dedicated incident command desk, and direct feedback to the WMAS Medical Director of all coroner cases, we do not believe we have missed any potentially eligible cases. However, it is possible that patients with methaemoglobinaemia from other causes, with potential to benefit from treatment with methylthioninium chloride, were not identified due to the nature of prehospital medicine precluding gold-standard diagnosis of raised MetHb levels. Additionally, we do not report patient data confirming the diagnosis of methaemoglobinaemia using blood sampling, as WMAS lack suitable data sharing agreements with the multiple receiving hospitals within the geographical area.

### Potential PGD changes

The current PGD may warrant revision. A PGD provides the legal framework for administration by non-prescribers; however, this requires strict adherence to the written protocol and legally precludes case-by-case clinical direction from external sources like the NPIS. Within this legal constraint, the dosing range could be aligned more closely with TOXBASE advice, as concerns regarding serotonin toxicity are rare at doses under 7 mg/kg and the principle of direct collaboration with NPIS on national treatment guidelines is well-established.^6 13^ A key consideration remains the reliance on non-invasive CO-Oximetry, as its accuracy for the high readings that guide treatment is uncertain.^14^,^16^ The PGD also excludes use in cardiac arrest, which is consistent with TOXBASE but differs from American Heart Association guidelines, which mention the drug for life-threatening toxicity but note a lack of evidence to guide its specific use during cardiac arrest.^17 18^

### The need for further research

Given that several UK ambulance services are now considering similar models, a national rollout is a realistic prospect. However, there is a risk that enthusiasm for this novel treatment could outpace the evidence, leading to premature adoption. Treatment guidelines require a firm foundation in clinical evidence, with an onus on guideline authors to acknowledge when existing evidence is insufficient.^19 20^

Therefore, before widespread implementation, a formal study is essential to build this evidence base. We suggest that an appropriate evaluation could take the form of a stepped wedge cluster randomised trial. Designing and delivering such a study would require significant expertise encompassing emergency medicine, clinical toxicology and clinical trials methodology.^21^,^23^ Proceeding with an unevaluated national roll-out risks not only the inefficient allocation of significant resources but also potential patient harm from an intervention that has not yet been proven safe and effective in the prehospital setting.

## Conclusion

This evaluation demonstrated that it is feasible to deliver prehospital methylthioninium chloride for probable sodium nitrite-induced methaemoglobinaemia in the UK using specialised paramedics, and that this enables the prehospital treatment of methaemoglobinaemia at relatively low cost.

Findings from this evaluation can inform decisions regarding adoption of methylthioninium chloride in ambulance trusts across the UK. The use of prehospital methylthioninium chloride for the treatment of methaemoglobinaemia currently lacks sufficient evidence to support adoption without further evaluation. A stepped wedge cluster randomised trial would allow for the real-world impact to be clearly understood.

## Data Availability

No data are available.

## References

[1] Thanacoody HKR, Bateman DN, Jefferson RD, Thomas SHL (2014). OxfordDesk Reference: Toxicology.

[2] Howland MA, Nelson LS, Howland MA, Lewin NA (2019). Goldfrank’s toxicologic emergencies.

[3] Hikin LJ, Ho J, Morley SR (2023). Sodium nitrite poisoning: A series of 20 fatalities in which post-mortem blood nitrite and nitrate concentrations are reported. Forensic Sci Int.

[4] Mack KA, Kaczkowski W, Sumner S (2024). Special Report from the CDC: Suicide rates, sodium nitrite-related suicides, and online content, United States. J Safety Res.

[5] Schölin L, Humphries C, Eddleston M (2025). De-siloing substance misuse and self-harm research through integrated public health and emergency medicine. Lancet Public Health.

[6] Bradberry A (2023). EAPCCT fellows’ webinar: methemoglobinaemia: diagnosis and treatment. https://eapcct.org/fellows/.

[7] TOXBASE (2022). Sodium nitrite. https://www.toxbase.org/poisons-index-a-z/s-products/sodium-nitrite/.

[8] Humphries C, Eddleston M, Dear J (2023). The emergency treatment of poisoning. Medicine (Baltimore).

[9] Brown SN, Kumar DS, James C (2019). JRCALC Clinical Guidelines.

[10] Garcia-Galindo CA, Pepin LC, Olives TD (2024). Massive Sodium Nitrite Overdose: A Case for Prehospital Methylene Blue. Prehosp Emerg Care.

[11] Medicines & Healthcare products Regulatory Agency (2017). Patient group directions: who can use them. https://www.gov.uk/government/publications/patient-group-directions-pgds/patient-group-directions-who-can-use-them.

[12] Harnett JT, Vithlani S, Sobhdam S (2021). National audit of antidote stocking in UK emergency departments. *Eur J Hosp Pharm*.

[13] Blundell M, Gill R, Thanacoody R (2024). Joint RCEM and NPIS best practice guideline: assessment and management of acute opioid toxicity in adults in the emergency department. Emerg Med J.

[14] Masimo SpMet product information. https://professional.masimo.co.uk/technology/co-oximetry/spmet/.

[15] US FDA (2013). Pulse oximeters – premarket notification submissions. https://www.fda.gov/media/72470/download.

[16] Zaouter C, Zavorsky GS (2012). The measurement of carboxyhemoglobin and methemoglobin using a non-invasive pulse CO-oximeter. Respir Physiol Neurobiol.

[17] Cooper GA, Bradberry SM, Sandilands EA (2023). Abstract presented at: 43rd International Congress of the European Association of Poisons Centres and Clinical Toxicologists (EAPCCT).

[18] Lavonas EJ, Akpunonu PD, Arens AM (2023). 2023 American Heart Association Focused Update on the Management of Patients With Cardiac Arrest or Life-Threatening Toxicity Due to Poisoning: An Update to the American Heart Association Guidelines for Cardiopulmonary Resuscitation and Emergency Cardiovascular Care. Circulation.

[19] Humphries C (2025). Clinical guidelines: what drives local adoption?. Emerg Med J.

[20] Humphries C, Boyle AA, France J (2024). Understanding RCEM Best Practice Guidelines. Emerg Med J.

[21] Humphries C, Clarke E, Eddleston M (2024). Study protocol: hisnap trial – a multi-centre, randomised, open label, blinded end-point, safety and efficacy trial of conventional (300mg/kg) versus higher doses of acetylcysteine (450mg/kg and 600mg/kg) in patients with paracetamol overdose in the united kingdom. *Pharmacology and Therapeutics*.

[22] Humphries C, Addison M, Aithal G (2024). Macrophage Therapy for Acute Liver Injury (MAIL): a study protocol for a phase 1 randomised, open-label, dose-escalation study to evaluate safety, tolerability and activity of allogeneic alternatively activated macrophages in patients with paracetamol-induced acute liver injury in the UK. BMJ Open.

[23] Humphries C, Roberts G, Taheem A (2023). SNAPTIMED study: does the Scottish and Newcastle Antiemetic Protocol achieve timely intervention and management from the emergency department to discharge for paracetamol poisoning?. Emerg Med J.

